# Is Distal Revascularization with Interval Ligation Still the Gold Standard? A Comparative Meta-Analysis and Meta-Regression of Surgical Treatments of Dialysis Access–Associated Steal Syndrome

**DOI:** 10.34067/KID.0000001252

**Published:** 2026-06-16

**Authors:** Maria Lourdes Del Río-Solá, Sandra Pérez-Fernández, Marina Jimenez-Caja, Clara De la Torre-Casaseca, Sergio Asensio-Rodriguez

**Affiliations:** 1 Department of Surgery, University of Valladolid, Valladolid, Spain; 2 Department of Vascular Surgery, University Clinical Hospital of Valladolid, Valladolid, Spain; 3 Department of Health Sciences, Universidad Europea Miguel de Cervantes, Valladolid, Spain

**Keywords:** arteriovenous access, arteriovenous fistula, arteriovenous graft, hemodialysis access

## Abstract

**Key Points:**

- Surgical treatment of dialysis access–associated steal syndrome achieves high symptom resolution rates, approaching 90% across observational studies.
- No significant differences were detected between distal revascularization with interval ligation and proximalization of the arterial inflow in symptom relief, complications, or primary patency.
- Current evidence supports an individualized, anatomy-driven approach rather than a single gold standard.

**Background:**

Distal revascularization with interval ligation (DRIL) is widely regarded as the gold standard for dialysis access–associated steal syndrome, yet high-level comparative evidence remains scarce. We performed a systematic review and meta-analysis to evaluate whether DRIL truly outperforms alternative reconstructive strategies, particularly proximalization of the arterial inflow (PAI).

**Methods:**

A Preferred Reporting Items for Systematic Reviews and Meta-Analyses‑compliant systematic search identified observational studies reporting outcomes after DRIL, PAI, or revision using distal inflow for dialysis access–associated steal syndrome. The review protocol was prospectively registered in the International Prospective Register of Systematic Reviews (CRD420261329250) before data extraction and study selection. The primary end point was symptom resolution. Secondary outcomes included primary patency and postoperative complications. Subgroup analyses and meta-regression were performed to explore technique-related differences and heterogeneity.

**Results:**

Eight studies were included. Overall symptom resolution reached 92% (95% confidence interval, 87% to 96%), with moderate heterogeneity (I^2^=58%). DRIL achieved a pooled resolution rate of 91%, while PAI demonstrated 93%, with no significant between-technique difference (*P* = 0.62). No statistically significant difference in complication rates was detected between DRIL and PAI (*P* = 0.56). No statistically significant difference in primary patency was detected between techniques (*P* = 0.34). Meta-regression did not identify publication year or study size as significant moderators. Influence diagnostics confirmed robustness of pooled estimates.

**Conclusions:**

The available observational evidence—derived exclusively from small retrospective cohorts predominantly at serious risk of bias—did not demonstrate a statistically significant difference between DRIL and PAI in symptom resolution, complications, or primary patency. These results should not be interpreted as equivalence or as justification for abandoning DRIL as a cornerstone technique. Rather, they reflect the limitations of the current evidence base. In the absence of randomized data, an individualized, anatomy-driven approach is recommended, and prospective comparative studies are urgently needed.

## Visual Abstract

**Figure:**
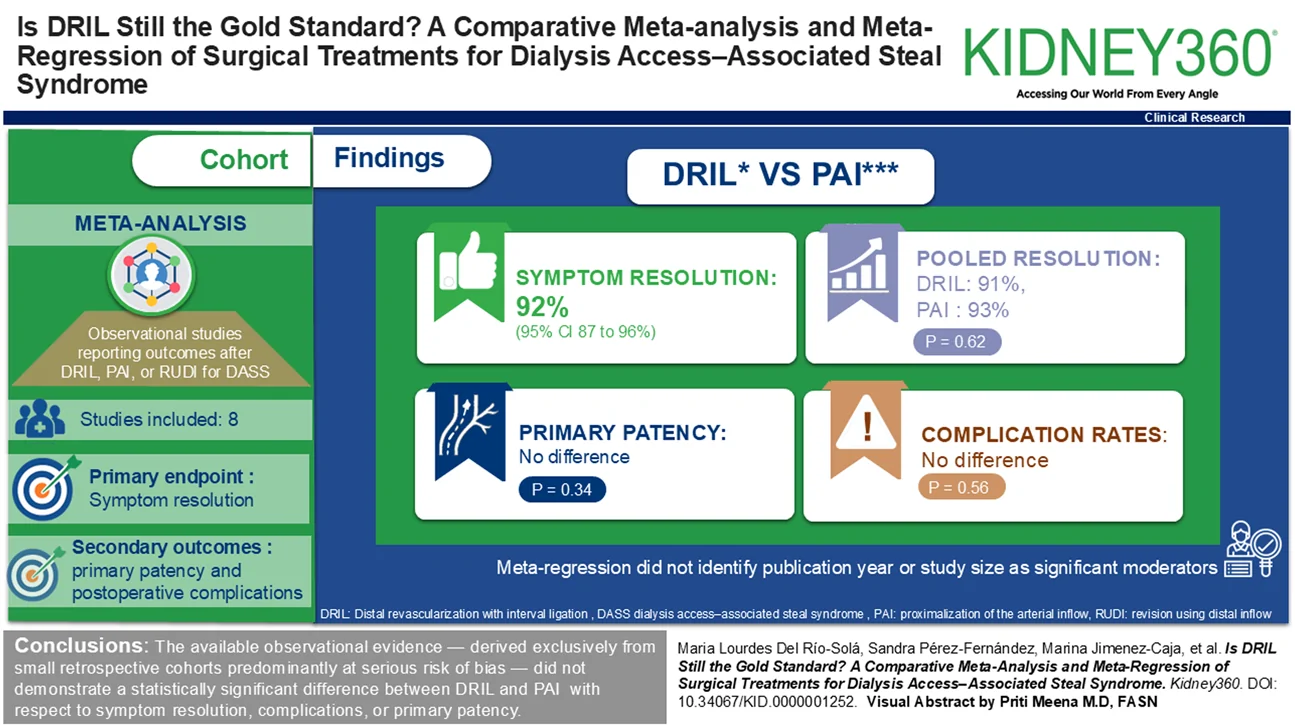

## Introduction

The growing number of patients requiring hemodialysis has increased the demand for durable arteriovenous access, whereas dialysis access–associated steal syndrome (DASS) remains a serious complication with an incidence of 3%–8%.^1–3^ DASS arises from a combination of reduced arterial inflow, preferential diversion into the access circuit, insufficient collateralization, and underlying arterial disease, leading in severe patient cases to rest pain, neurologic deficits, or tissue loss requiring urgent intervention.^1,4^

Surgical management aims to relieve ischemia while preserving access. Ligation is effective but sacrifices the access,^2^ whereas flow-reduction techniques, such as banding, have shown inconsistent outcomes.^5^ Distal revascularization with interval ligation (DRIL) is widely considered the gold standard, providing high rates of symptom resolution and access patency.^1,6^ Alternative approaches include revision using distal inflow (RUDI), which reduces access flow, and proximalization of the arterial inflow (PAI), which restores hemodynamic balance without arterial ligation.^5,7,8^ Although promising, evidence for these techniques is largely observational.

Comparative data between DRIL, RUDI, and PAI remain limited, with most studies being retrospective and heterogenous, preventing definitive conclusions regarding their relative effectiveness.

The objective of this study was therefore to perform a systematic review and comparative meta-analysis of surgical treatments of DASS, with DRIL versus PAI as the primary quantitative comparison, assessing symptom resolution, complication rates, and primary patency. Available evidence on RUDI is presented descriptively. Specifically, we sought to determine whether DRIL remains the optimal strategy or whether available observational evidence suggests differences between PAI and DRIL in contemporary practice, while also contextualizing the role of RUDI within the current therapeutic armamentarium.

## Methods

### Study Design and Protocol Registration

This systematic review and comparative meta-analysis was conducted in accordance with the Preferred Reporting Items for Systematic Reviews and Meta-Analyses guidelines. The study protocol was prospectively developed before data extraction and statistical analysis to minimize bias and ensure methodological transparency. The review protocol was submitted for registration in the International Prospective Register of Systematic Reviews (CRD420261329250) before data extraction and study selection.

Although the protocol included DRIL, PAI, and RUDI as eligible interventions, quantitative meta-analysis was restricted to DRIL and PAI. No primary study identified in our systematic search reported RUDI outcomes in a format amenable to quantitative pooling. Therefore, RUDI data are presented descriptively.

### Eligibility Criteria

Studies were considered eligible if they included adult patients undergoing surgical treatment of DASS and reported outcomes after DRIL, RUDI, or PAI. Both comparative and noncomparative cohort studies were included, provided they reported extractable data on at least one of the predefined clinical end points. Randomized controlled trials, prospective cohort studies, and retrospective observational studies were eligible. Case reports, small case series including fewer than five patients, editorials, conference abstracts without full text, and experimental studies were excluded.

### Search Strategy

A comprehensive and systematic literature search was conducted in PubMed/MEDLINE, Embase, Cochrane Library, and Web of Science from database inception through the final search date (January 15, 2025). The search strategy combined Medical Subject Headings with relevant free-text terms addressing DASS and the surgical techniques evaluated in this study. Specifically, search terms included variations of “dialysis access steal syndrome,” “hemodialysis access ischemia,” and “access-induced ischemia,” together with procedure-specific terms, such as “DRIL,” “distal revascularization interval ligation,” “RUDI,” “revision using distal inflow,” “PAI,” and “proximalization of arterial inflow.” These terms were combined using appropriate Boolean operators to maximize sensitivity while maintaining specificity. In addition to the electronic search, the reference lists of all included studies were manually reviewed to identify any additional relevant publications that may not have been captured through database searching.

### Study Selection

Two independent reviewers screened titles and abstracts for relevance. Full-text articles were subsequently assessed for eligibility according to predefined inclusion and exclusion criteria. Disagreements were resolved through discussion and consensus. A Preferred Reporting Items for Systematic Reviews and Meta-Analyses flow diagram was constructed to document the study selection process.

### Data Extraction

Data were independently extracted by two investigators using a predefined and standardized data collection form to ensure consistency and minimize transcription errors. For each eligible study, information regarding the first author, year of publication, country of origin, and study design was recorded. In addition, detailed clinical data were collected, including total sample size, type of surgical intervention performed (DRIL, RUDI, or PAI), number of patients experiencing symptom improvement, number without improvement, rates of access preservation, reported patency outcomes, and documented complications. Follow-up duration was also extracted whenever available to allow appropriate interpretation of outcome durability. In cases of incomplete or unclear reporting, corresponding authors were contacted to obtain clarification or additional data when feasible.

### Outcomes

The primary outcome of interest was overall symptom improvement, defined as complete or partial resolution of ischemic symptoms at the latest available follow-up reported by each study. Secondary outcomes included access preservation (defined as absence of thrombosis or abandonment of the original arteriovenous access during the reported follow-up period, regardless of the need for secondary interventions to maintain function), primary and secondary patency when reported, reintervention rates, major complications, and limb loss. Outcomes were extracted at the latest reported follow-up time point available in each study. Given the heterogeneity in follow-up duration across studies (range, 3–36 months), direct comparison of patency data should be interpreted with caution.

### Risk of Bias Assessment

The methodological quality of included studies was independently assessed by two reviewers using the Risk Of Bias In Non-randomized Studies - of Interventions (ROBINS-I) tool, appropriate for nonrandomized observational studies, because no randomized trials were identified. The ROBINS-I tool evaluates seven domains of bias (confounding, participant selection, intervention classification, deviations from intended interventions, missing data, outcome measurement, and selective reporting), each rated as low, moderate, serious, or critical. Overall risk of bias was determined by the highest risk domain, with studies classified as serious if at least one domain was rated as such. Disagreements were resolved by consensus or consultation with a third reviewer.

### Statistical Analyses

Given the expected clinical and methodological heterogeneity among studies, pooled analyses were performed using a random-effects model according to the DerSimonian–Laird method. Because the primary end point consisted of proportions (symptom improvement rates), event rates were transformed using logit transformation before pooling to stabilize variances. Between-study heterogeneity was assessed using the I^2^ statistic, with values of 25%, 50%, and 75% representing low, moderate, and high heterogeneity, respectively.

Subgroup analyses were conducted according to surgical technique (DRIL and PAI). RUDI could not be included in subgroup analyses because no primary study identified in our systematic search reported RUDI outcomes in a format amenable to quantitative pooling; therefore, RUDI data are presented descriptively in a dedicated subsection of the Results section. Sensitivity analyses were performed by excluding small studies and those with lower methodological quality. Publication bias was explored through visual inspection of funnel plots and Egger regression test. All statistical analyses were performed using SPSS version 29.0 (IBM Corp., Armonk, NY), with additional cross-validation performed using Python to ensure analytical robustness. A two-sided *P* < 0.05 was considered statistically significant.

## Results

### Study Selection and Study Characteristics

Eight studies met the predefined inclusion criteria and were included in the quantitative synthesis (Figure 1). All included investigations were retrospective or prospective observational cohorts, and no randomized controlled trials were identified. The studies were published between 2002 and 2024 and showed variability in sample size, baseline characteristics, surgical technique distribution, and follow-up duration. Four studies evaluated DRIL, five studies assessed PAI, and one study contributed data to both techniques. Although reporting standards and outcome definitions were not fully uniform, all studies provided extractable data for symptom resolution. Complications and primary patency were reported only in subsets of cohorts and were therefore analyzed separately (Table 1). The principal findings of each included study, including rates of symptom resolution, patency outcomes, and complication profiles, are summarized in Table 2.

**Figure 1:**
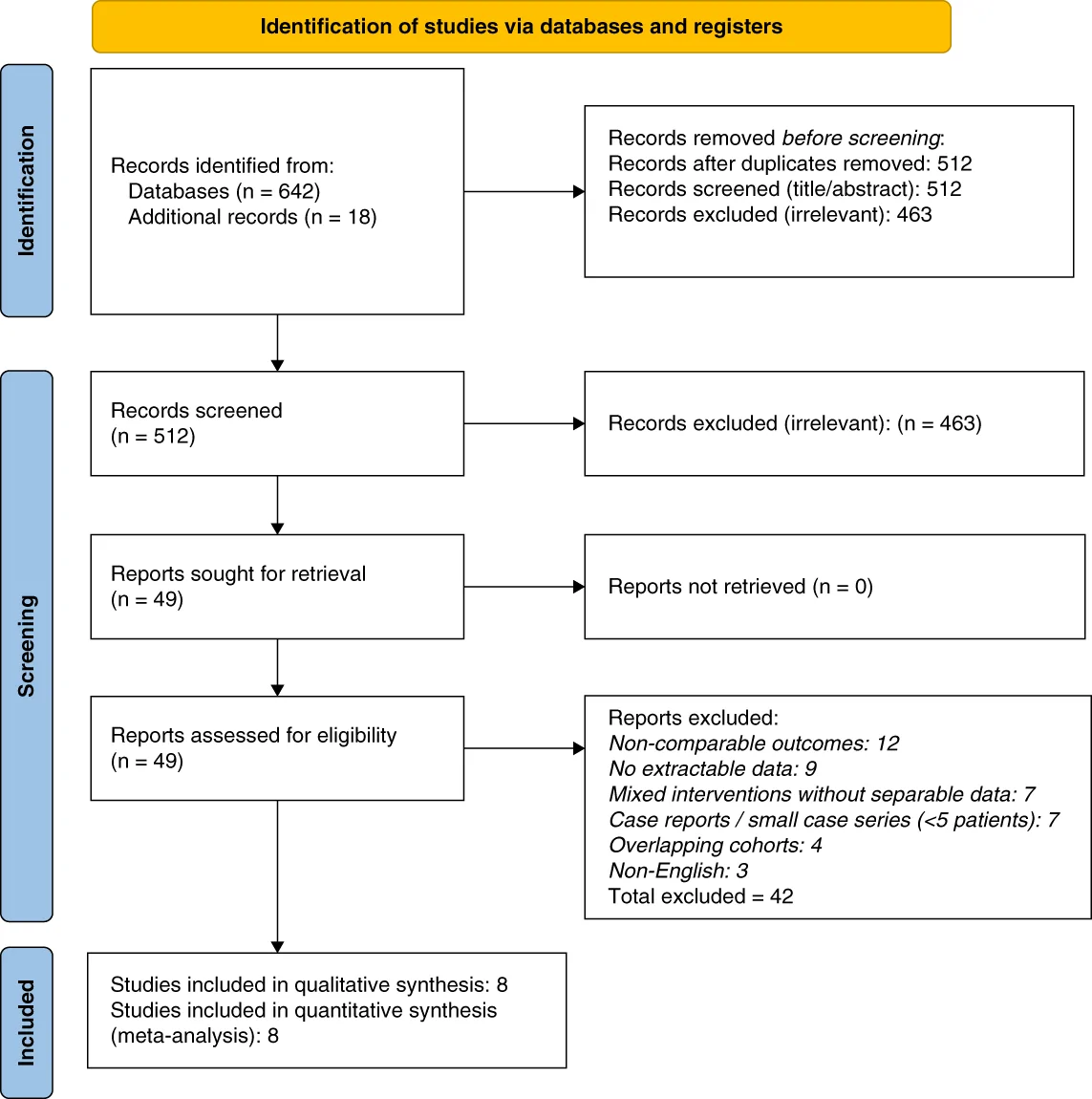
**PRISMA flow diagram.** Flow diagram illustrating the identification, screening, eligibility, and inclusion process according to PRISMA 2020 guidelines. A total of 660 records were identified through database searching and manual screening. After removal of 148 duplicates, 512 titles and abstracts were screened, leading to 49 full-text articles assessed for eligibility. Forty-one studies were excluded for predefined reasons, and eight observational studies were ultimately included in the qualitative and quantitative syntheses. PRISMA, Preferred Reporting Items for Systematic Reviews and Meta-Analyses.

**Table 1:** Baseline characteristics of included studies

Study	Year	Country	Design	*N* (Patients)	Technique	Mean Age (yr)	% Female	% DM	Follow-Up	DASS Severity at Baseline	Access Type	Inflow Artery
Knox *et al.*^9^	2002	United States	Retrospective cohort	55 procedures (52 patients)	DRIL	61	67	92	Mean 16 mo (range, 1–67 mo)	Rest pain 52%; tissue loss 38%; neurologic loss 8%; pain on hemodialysis 2%	Brachiocephalic AVF 37%; upper arm ePTFE graft 37%; forearm ePTFE graft 23%; basilic transposition 3%	Brachial artery (all patients)
Zanow *et al.*^8^	2006	Germany	Prospective cohort	30	PAI	67±9	43	80	Mean 26 mo (range, 3–61 mo)	Rest pain 33%; tissue loss 37% (ulcers 20%, gangrene 17%); pain on dialysis 17%; paresthesia 13%	Brachiocephalic AVF 17; brachiobasilic 2; radial-cephalic 4; prosthetic brachioaxillary 4 (mixed)	Axillary artery (distal or proximal, per anatomy)
Huber *et al.*^6^	2008	United States	Retrospective cohort	64 procedures (61 patients)	DRIL	58±13	62	72	Mean 12 mo (range, 0–66 mo); up to 5 yr	Pain 25%; paresthesia 34%; motor dysfunction 24%; tissue loss 17%	Brachiocephalic AVF 46%; brachiobasilic AVF 31%; brachioaxillary translocated femoral vein 20%; prosthetic 3%	Brachial artery (all patients)
Thermann *et al.*^7^	2010	Germany	Prospective cohort	40 procedures (38 patients)	PAI	70 (range, 54–86)	42	69	6–40 mo (median 19 mo)	Stage 2 (pain, no lesions) 8%; stage 3 (small acral lesions) 82%; stage 4 (extended lesions) 10%	Elbow fistulas 83% (brachiocephalic/brachiobasilic); wrist fistulas 17%	Axillary artery (distal/proximal per anatomy)
Gupta *et al.*^10^ ^a^	2011	United States	Retrospective cohort	70 procedures (114 ISS patients total)	DRIL/RUDI/PAI	65 (range, 20–90)	66	61	30-d outcomes; median 72 d to intervention	Moderate–severe ISS: grade 2 (rest pain/neurologic) and grade 3 (gangrene/motor loss)	Brachial origin AVF 69%; prosthetic graft 16%; radial-cephalic 13%	Brachial (DRIL); radial/ulnar (RUDI); axillary (PAI)
Leake *et al.*^5^	2015	United States	Retrospective cohort	218 DASS episodes (201 patients)	DRIL, PAI, RUDI, banding, ligation, DRAL	65 (range, 19–90)	62	68	Median 23 d to first follow-up (30-d outcomes)	Grade 2 (rest pain±neurologic) 48%; grade 3 (motor dysfunction or tissue loss) 52%	Autologous AVF 80%; prosthetic graft 19%; brachial artery-based 87%; cephalic outflow 57%, basilic 29%	Variable per technique: brachial (DRIL 95%); radial/ulnar (RUDI); axillary (PAI)
Alie-Cusson *et al.*^11^	2021	United States	Retrospective cohort	89	DRIL	60 (range, 23–88)	60	76	Up to 180 d (6 mo patency reported)	Class 2 (mild) 20%; class 3 (moderate) 55%; class 4 (severe) 25%	AVF 80%: brachial-cephalic 55%, brachial-basilic 25%; AVG 20% (brachial-axillary 18%)	Brachial artery (all DRIL cases); GSV conduit 93%
Bachmann *et al.*^12^	2024	United States	Retrospective cohort	64	PAI	65±15 (range, 27–92)	59	80	Mean patency 45 mo; primary patency reported at 12 and 24 mo	Grade 1 25%; grade 2 16%; grade 3 33%; grade 4 26%	AVF 95%: brachiobasilic 72%, brachiocephalic 14%, proximal radial 8%; AVG 5%	Axillary artery (4–7 mm tapered ePTFE graft in majority)

AVF, arteriovenous fistula; AVG, arteriovenous graft; DASS, dialysis access–associated steal syndrome; DM, diabetes mellitus; DRAL, distal radial artery ligation; DRIL, distal revascularization with interval ligation; ePTFE, expanded polytetrafluoroethylene; GSV, great saphenous vein; ISS, ischemic steal syndrome; PAI, proximalization of the arterial inflow; RUDI, revision using distal inflow.

a Gupta *et al.*^10^ reported outcomes for distal revascularization with interval ligation (*n*=21), revision using distal inflow (*n*=4), and proximalization of the arterial inflow (*n*=3) as separate cohorts within a single study. This study is counted once in the total number of included studies (*n*=8) but contributes data to multiple technique subgroups in the quantitative analysis.

**Table 2:** Main findings of included studies on surgical management of dialysis access–associated steal syndrome

Study	Technique	Symptom Resolution	Primary Patency	Complications	Authors' Main Conclusion
Knox *et al.*,^9^ 2002	DRIL	90% substantial or complete relief (47/52 patients); three patients required access ligation for failed DRIL	Bypass primary patency 80% at 48 mo; access primary patency 83% at 12 mo, 71% at 36 mo	Wound complications 9% (5/55); no major perioperative complications; no limb loss	DRIL is durable and effective, reliably achieving symptom relief and access preservation at midterm follow-up
Zanow *et al.*,^8^ 2006	PAI	84% complete resolution; 16% significant improvement; 100% overall improvement rate; no access ligation required	Primary patency 87% at 1 yr, 67% at 3 yr; secondary patency 90% at 1 yr, 78% at 3 yr	No early postoperative complications; graft thrombosis in follow-up managed by thrombectomy; graft infection 1 patient (3%) at 19 mo	PAI preserved native arterial continuity and demonstrated favorable symptom relief and patency in this cohort
Huber *et al.*,^6^ 2008	DRIL	78% complete resolution; residual paresthesia 13%; pain 5%; tissue loss 4%; motor 2%. Access ligation required in 1 patient	Bypass primary patency 77% at 1 yr, 74% at 3 yr, 71% at 5 yr; secondary patency 81%, 76%, 76%	Overall complication rate 22%; wound 14%; respiratory 3%; vascular 3%; cardiac 2%; perioperative mortality 3%	DRIL safely and effectively relieves severe access-related hand ischemia; midterm bypass patency is durable; long-term surveillance warranted
Thermann *et al.*,^7^ 2010	PAI	Immediate clinical success 90% (36/40); permanent success 85% (34/40); 3 patients required early access closure due to failure	Primary patency 77% at 6 mo, 62% at 12 mo, 42% at 24 mo; secondary patency 75% at 18 mo; heparinized grafts: 86% at 6 mo, 75% at 12 mo	Graft thrombosis in 12 patients (30%); graft explantation in 7 patients; poor results in stage 4 (extended tissue loss)	PAI provides good long-term clinical results in DASS stage 3; patency improvement needed; stage 4 should be considered for access closure
Gupta *et al.*,^10^ 2011^a^	DRIL/RUDI/PAI	DRIL 80% (20/25 patients); RUDI 100% (3/3); PAI 100% (3/3); banding 38%; ligation 0% access preservation	Not formally reported (30-d outcomes study); 100% fistula preservation with DRIL, RUDI, and PAI	Banding: 62% overall failure rate; DRIL significantly better than banding (*P* < 0.05); RUDI/PAI: 0% complications in small samples	RUDI and PAI highly successful in small numbers; DRIL effective but not uniformly; banding has unacceptably high failure and reintervention rates
Leake *et al.*,^5^ 2015	DRIL, RUDI, PAI, banding, ligation, DRAL	DRIL 98%; RUDI 95%; PAI 100%; banding 75%; ligation 93%; DRAL 100%	Access preservation: DRIL 100%; RUDI 95%; PAI 100%; banding 89%; ligation 0%. Patency rates not formally reported	30-d complications: DRIL 7%; ligation 8%; RUDI 37%; banding 47%; PAI 44%. Banding highest thrombosis; RUDI highest bleeding risk. Mortality: DRIL 7%	DRIL offers superior access preservation and fewer complications versus banding/RUDI; recommended as preferred procedure for functional autologous AVF with DASS
Alie-Cusson *et al.*,^11^ 2021	DRIL	Complete resolution 70% (62/89); partial 26% (23/89); no improvement 4% (4/89); overall improvement 96%	Bypass primary patency 93% at 6 mo; bypass secondary 100% at 6 mo. Access primary patency 79% at 6 mo; access secondary 94% at 6 mo	Wound infections 24% (21 patients, 24 cases); 71% at saphenectomy site; 52% required surgical debridement; 5 patients needed reoperation for DASS	DRIL highly effective for DASS with excellent short-term patency; wound infection rate is a significant concern, especially at saphenectomy site
Bachmann *et al.*,^12^ 2024	PAI	Complete resolution 86% (55/64); partial 8% (5/64); no resolution 3% (2/64); total improvement 94%	Primary patency: 94% at 1 mo, 81% at 12 mo, 71% at 24 mo. Secondary patency: 97% at 1 mo, 87% at 12 mo, 84% at 24 mo	30-d complication rate 11% (7/64): thrombosis 8% (5/64), thrombosis+infection 2% (1/64), upper extremity swelling 2% (1/64). No limb loss or tissue loss post-PAI	PAI was reported as a reliable option for HAIDI with favorable safety and efficacy outcomes
**Contextual reference—not included as a primary study in this meta-analysis**					
Kordzadeh *et al.*,^13^ 2018^b^	RUDI	82% success rate (106/130; 95% CI, 74.4% to 89.6%) over median 12 mo follow-up; 11% required access ligation for ongoing DASS	Median patency 12 mo (IQR, 1–40); AVF thrombosis post-RUDI 8% (10/130)	Overall morbidity 3% (4/130): single-digit amputation 2% (2/130); ischemic neuropathy 1% (1/130); AVF rupture/hematoma 1% (1/130). Mortality 0%	RUDI is feasible and effective for DASS grades 1–3; best outcomes with brachiocephalic AVF; grade 4 associated with worst outcomes

AVF, arteriovenous fistula; CI, confidence interval; DASS, dialysis access–associated steal syndrome; DRAL, distal radial artery ligation; DRIL, distal revascularization with interval ligation; HAIDI, hemodialysis access‑induced distal ischemia; IQR, interquartile range; PAI, proximalization of the arterial inflow; ROBINS-I, Risk Of Bias In Non-randomized Studies - of Interventions; RUDI, revision using distal inflow.

a Gupta *et al.*^10^ reported outcomes for distal revascularization with interval ligation (*n*=21), revision using distal inflow (*n*=4), and proximalization of the arterial inflow (*n*=3) as separate cohorts within a single study; this study is counted once in the total number of included studies (*n*=8) but contributes data to multiple technique subgroups.

b The study by Kordzadeh *et al.*^13^ is a systematic review and was not included as a primary study in this meta-analysis because only primary observational or interventional studies were eligible. This reference is cited solely as contextual background to summarize available revision using distal inflow evidence and was not included in the qualitative or quantitative synthesis nor assessed using the ROBINS-I tool.

### Risk of Bias Assessment

Given that all included studies were retrospective observational cohorts, the overall risk of bias was primarily driven by nonrandom allocation of surgical technique—with potential confounding by indication—heterogeneity in reporting of baseline clinical features and follow-up and variability in outcome definitions. Nevertheless, outcome reporting for the primary end point (symptom resolution) was sufficiently consistent to allow pooling, and influence diagnostics did not indicate that any single study dominated the overall estimate. According to the ROBINS-I tool, most of the studies were judged to be at serious risk of bias and no study achieved a low risk of bias classification. The overall certainty of evidence is therefore limited and should be interpreted with caution (Table 3).

**Table 3:** Assessment of risk of bias in nonrandomized intervention studies included in the meta-analysis (ROBINS-I tool)

Study	Bias Due to Confounding	Selection of Participants	Classification of Interventions	Deviations from Intended Interventions	Missing Data	Measurement of Outcomes	Selective Reporting	Overall Risk of Bias
Knox *et al.*^9^ (2002)	Serious	Moderate	Low	Low	Moderate	Moderate	Moderate	Serious
Zanow *et al.*^8^ (2006)	Moderate	Moderate	Low	Low	Moderate	Moderate	Moderate	Moderate–Serious
Huber *et al.*^6^ (2008)	Serious	Moderate	Low	Low	Moderate	Moderate	Moderate	Serious
Thermann *et al.*^7^ (2010)	Moderate	Moderate	Low	Low	Moderate	Moderate	Moderate	Moderate–Serious
Gupta *et al.*^10^ (2011)	Serious	Moderate	Low	Moderate	Moderate	Moderate	Moderate	Serious
Leake *et al.*^5^ (2015)	Moderate	Moderate	Low	Moderate	Moderate	Moderate	Moderate	Serious
Alie-Cusson *et al.*^11^ (2021)	Serious	Moderate	Low	Low	Moderate	Moderate	Moderate	Serious
Bachmann *et al.*^12^ (2024)	Serious	Moderate	Low	Low	Moderate	Moderate	Moderate	Serious

Risk level key: low|moderate|serious|moderate–serious.

The overall risk-of-bias judgment was determined according to ROBINS-I guidance: The global assessment reflects the highest level of risk identified across any individual domain. A study was classified as Serious overall if at least one domain was judged as Serious. Studies with all domains rated as Moderate or below were categorized as Moderate–Serious. No study achieved a Low overall risk-of-bias rating, reflecting the observational and nonrandomized design of all included investigations.

Domain definitions (ROBINS-I): (*1*) Bias due to confounding: potential for preexisting differences between intervention groups to distort the effect estimate; (*2*) selection of participants: bias arising from how participants were identified and enrolled; (*3*) classification of interventions: misclassification of the intervention received; (*4*) deviations from intended interventions: departures from the assigned intervention during the study; (*5*) missing data: incomplete outcome data and its potential impact; (*6*) measurement of outcomes: bias in how outcomes were assessed; and (*7*) selective reporting: incomplete or selective presentation of results.

Note on revision using distal inflow evidence: The study by Kordzadeh *et al.*^13^ is a systematic review and pooled analysis of 11 observational cohorts (*n*=130 patients) and was not evaluated using ROBINS-I, which is designed for individual nonrandomized studies. The overall quality of evidence for revision using distal inflow as reported by Kordzadeh *et al.*^13^ is level 3 with a grade C recommendation (NICE grading), on the basis of Oxford CASP appraisal of the 11 included cohorts. The study by Kordzadeh *et al.*^13^ was not included as a primary study in this meta-analysis and is cited solely as contextual background; see Table 2, footnote b. CASP, Critical Appraisal Skills Programme; NICE, National Institute for Health and Care Excellence; ROBINS-I, Risk Of Bias In Non-randomized Studies - of Interventions.

### Symptom Resolution

Random-effects pooling across all included studies yielded a logit-transformed event estimate of 2.48 (95% confidence interval [CI], 1.90 to 3.07; *P* < 0.001) for symptom resolution (Figure 2A). After inverse logit transformation, this corresponded to an overall clinical improvement rate of approximately 92% (95% CI, 87% to 96%). Between-study heterogeneity was moderate (τ^2^=0.40; I^2^=58%), and Cochran Q confirmed statistically significant heterogeneity (Q=20.59; df=8; *P* = 0.01). When stratified by surgical technique, DRIL demonstrated a pooled logit estimate of 2.34 (95% CI, 1.36 to 3.32; *P* < 0.001), corresponding to a resolution rate of 91% (95% CI, 80% to 96%), whereas PAI yielded 2.62 (95% CI, 2.05 to 3.20; *P* < 0.001), corresponding to 93% (95% CI, 89% to 96%; Figure 2A). Direct comparison between techniques did not demonstrate a statistically significant difference (Q-between=0.24; df=1; *P* = 0.62).

**Figure 2:**
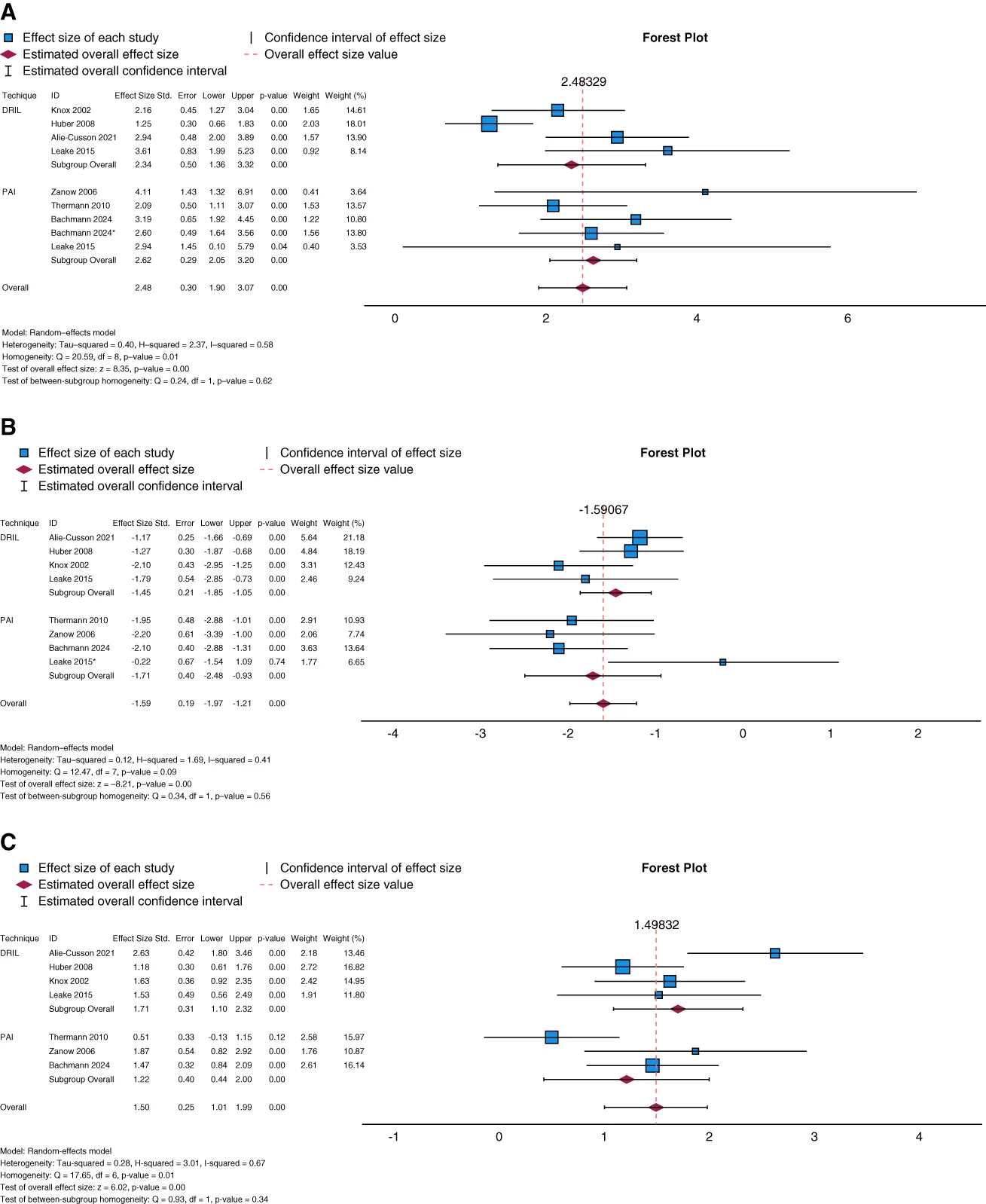
**Comparative clinical outcomes after surgical treatment of DASS.** (A) Forest plot of pooled symptom resolution rates after surgical treatment of DASS. (B) Forest plot of pooled postoperative complication rates. (C) Forest plot of pooled primary patency rates. All analyses were performed using random-effects models to account for anticipated clinical and methodological heterogeneity. For proportion-based outcomes, event rates were logit transformed before pooling to stabilize variances. Subgroup analyses comparing DRIL and PAI are presented within each panel. Effect sizes are expressed as logit-transformed proportions with 95% CIs. Between-study heterogeneity was assessed using I^2^ statistic. CI, confidence interval; DASS, dialysis access–associated steal syndrome; DRIL, distal revascularization with interval ligation; PAI, proximalization of the arterial inflow.

Random-effects meta-regression analyses (bubble plots) did not demonstrate a significant technique-driven effect (Figure 3A). Influence diagnostics using Galbraith (radial) plots showed that most of the studies lay within the 95% confidence limits without extreme outliers, supporting the stability of the pooled estimate (Supplemental Figure 1A).

**Figure 3:**
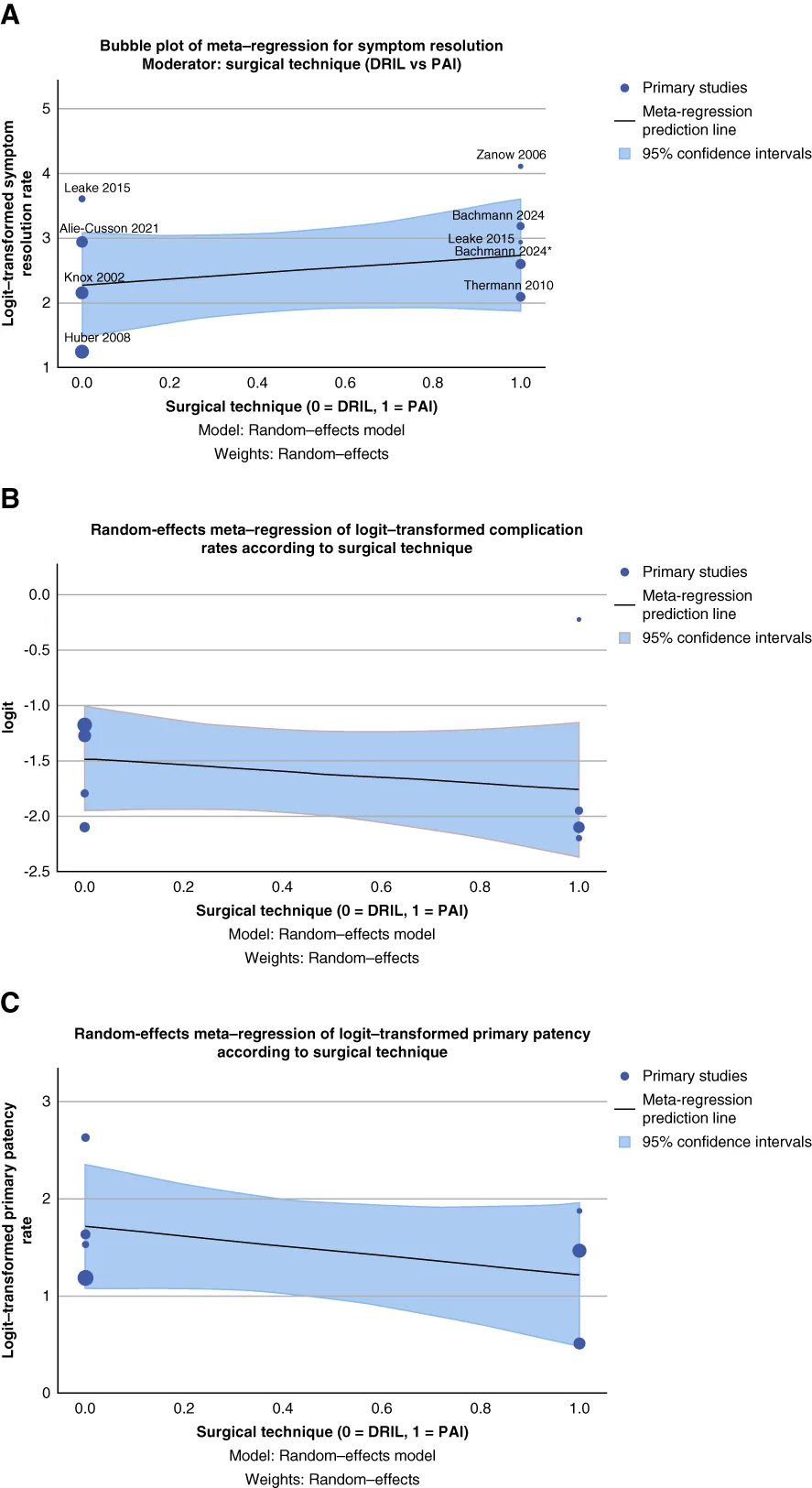
**Random-effects meta-regression analyses.** (A) Symptom resolution. (B) Postoperative complications. (C) Primary patency. Random-effects meta-regression analyses exploring the association between surgical technique and logit-transformed outcome rates. Surgical technique was coded as 0=DRIL and 1=PAI. Bubble size is proportional to the inverse variance study weight. The solid line represents the fitted meta-regression model, and the shaded area indicates the 95% confidence band.

Assessment of publication bias using funnel plot inspection did not reveal marked asymmetry (Figure 4A). Trim-and-fill analysis suggested three potentially missing studies; the adjusted pooled effect estimate was 2.188 (95% CI, 1.640 to 2.737), compared with the original estimate of 2.483 (95% CI, 1.900 to 3.066), while statistical significance was preserved (*P* < 0.001). Although minor publication bias cannot be fully excluded, the primary estimate seemed robust to this potential source of bias.

**Figure 4:**
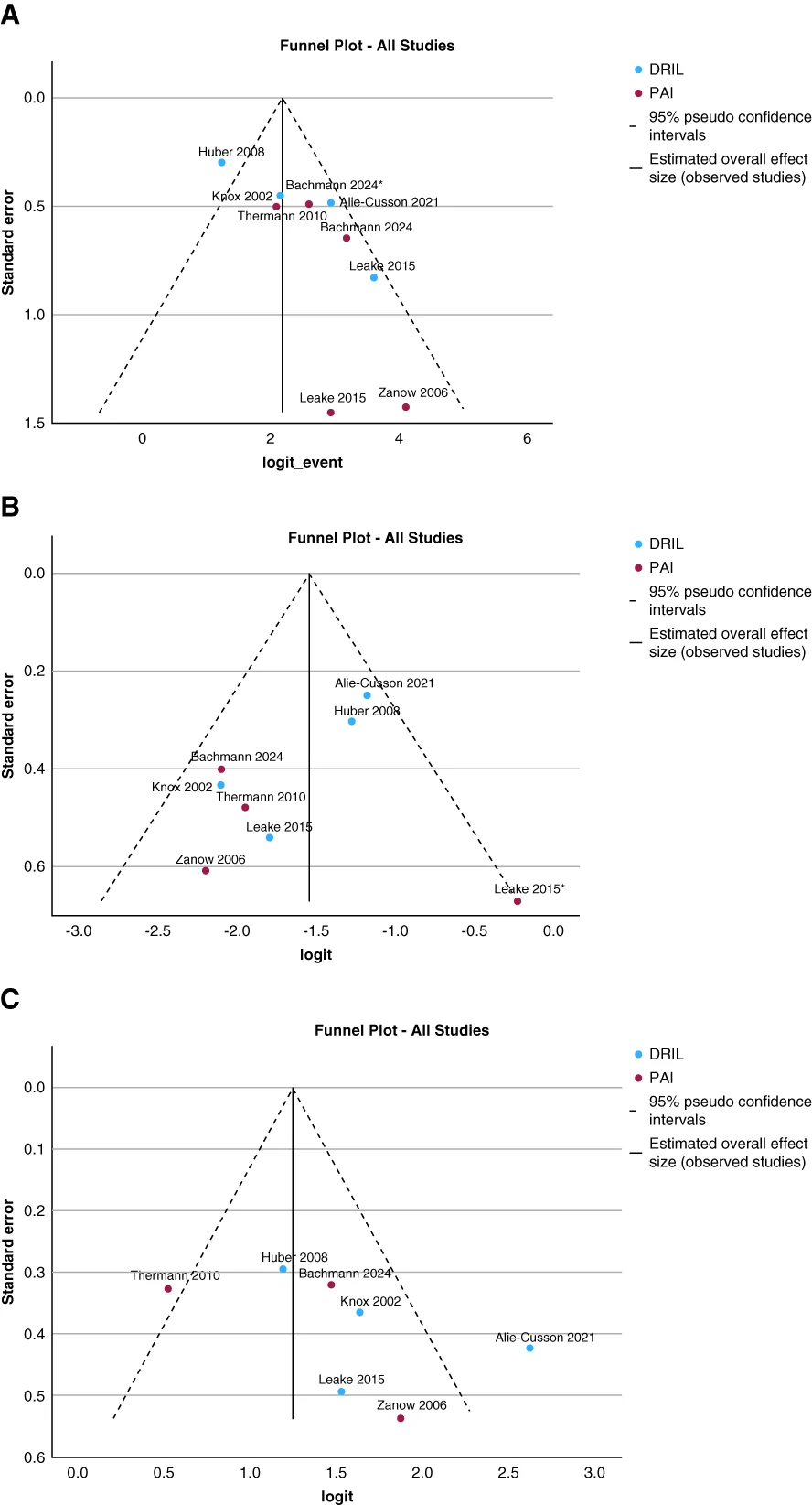
**Funnel plots assessing publication bias.** (A) Symptom resolution. (B) Postoperative complications. (C) Primary patency. Funnel plots were constructed to evaluate potential publication bias for each outcome. Logit-transformed effect sizes are plotted against their standard errors. In the absence of publication bias, studies are expected to be symmetrically distributed around the pooled random-effects estimate. Visual inspection was complemented by Egger regression test when applicable. Asymmetry may suggest small-study effects or selective reporting.

Random-effects meta-regression analyses are presented in Figure 3A. When publication year was entered as a continuous moderator, no statistically significant association was identified (*β*=0.052; 95% CI, −0.025 to 0.129; *P* = 0.15). Although the model attributed 39.2% of between-study heterogeneity to publication year (R^2^=39.2%), residual heterogeneity remained moderate (τ^2^=0.245; I^2^=43.8%).

Similarly, total sample size was not associated with treatment effect (*β*=−0.002; 95% CI, −0.043 to 0.040; *P* = 0.93). Study size explained none of the between-study variability (R^2^=0%), and residual heterogeneity remained substantial (τ^2^=0.480; I^2^=61.9%).

Influence diagnostics using Galbraith (radial) plots showed that most of the studies lay within the 95% confidence limits without extreme outliers, supporting the stability of the pooled estimate (Supplemental Figure 1A).

### Postoperative Complications

Pooled analysis demonstrated a logit effect estimate of −1.59 (95% CI, −1.97 to −1.21; *P* < 0.001), consistent with a low overall complication rate (Figure 2B). Between-study heterogeneity was moderate (τ^2^=0.12; I^2^=41%), and Cochran Q did not reach statistical significance (Q=12.47; df=7; *P* = 0.09). Subgroup analysis showed pooled logit estimates of −1.45 (95% CI, −1.85 to −1.05; *P* < 0.001) for DRIL and −1.71 (95% CI, −2.48 to −0.93; *P* < 0.001) for PAI (Figure 2B), without a significant between-group difference (Q-between=0.34; df=1; *P* = 0.56). Meta-regression bubble plots did not demonstrate a significant moderator effect of technique (Figure 3B). Galbraith diagnostics did not suggest the presence of a dominant outlier (Supplemental Figure 1B). Funnel plot inspection did not reveal clear asymmetry (Figure 4B).

### Primary Patency

Primary patency analysis yielded a pooled logit estimate of 1.50 (95% CI, 1.01 to 1.99; *P* < 0.001; Figure 2C). Heterogeneity was substantial (τ^2^=0.28; I^2^=67%), and Cochran Q confirmed significant variability (Q=17.65; df=6; *P* = 0.01). When stratified by technique, DRIL demonstrated a pooled logit estimate of 1.71 (95% CI, 1.10 to 2.32; *P* < 0.001), whereas PAI showed 1.22 (95% CI, 0.44 to 2.00; *P* < 0.001; Figure 2C). Despite numerical differences, the between-technique comparison was not statistically significant (Q-between=0.93; df=1; *P* = 0.34). Meta-regression bubble plots did not indicate a significant technique effect (Figure 3C). Galbraith plots demonstrated dispersion across studies without a dominant outlier (Supplemental Figure 1C). Funnel plot inspection did not show marked asymmetry (Figure 4C).

Interpretation of primary patency data across studies is further limited by substantial heterogeneity in follow-up duration (range: approximately 3–36 months across included cohorts) and nonuniform definitions of patency end points. These factors preclude direct cross-study comparisons of patency rates and should be considered when interpreting the pooled estimates.

### Secondary Outcomes: Access Preservation, Reintervention, and Limb Loss

These outcomes could not be meta-analyzed because of inconsistent reporting and heterogenous definitions, particularly for access preservation. Reported access preservation ranged from 78% to 100%. Reintervention rates (three studies) varied between 10% and 22%, while no cases of limb loss were reported (four studies). These findings are descriptive and should be interpreted with caution.

### RUDI: Descriptive Summary

No primary study identified in our systematic search reported RUDI outcomes in a format amenable to inclusion in the quantitative meta-analysis. Therefore, RUDI data are presented descriptively.

The following descriptive summary is based on the study by Kordzadeh *et al.*, a published systematic review cited as contextual background only and not included as a primary study in this meta-analysis. Evidence on RUDI for DASS derives exclusively from small single-center observational cohorts.

A systematic review pooling 11 studies and 130 patients reported a symptom resolution rate of 82% (95% CI, 74.4% to 89.6%) over a median follow-up of 12 months (interquartile range, 1–40 months). The most common access type was brachiocephalic arteriovenous fistula (AVF; 73.7%), and the most frequently used distal inflow artery was the radial artery (61.5%). Overall morbidity was 3%, with no mortality reported. AVF thrombosis after RUDI occurred in 7.6% of patient cases, and limb loss was rare (1.5%). The level of evidence was graded as III with a grade C recommendation. These results suggest that RUDI is a feasible access-preserving option in selected patients, particularly those with high-flow brachiocephalic AVF, although comparative conclusions relative to DRIL or PAI cannot be drawn from the available evidence.^13^

## Discussion

This systematic review and comparative meta-analysis evaluated contemporary surgical strategies for DASS, focusing on DRIL and PAI. Surgical correction of DASS achieved a high overall rate of symptom resolution (approximately 92%), consistent with prior reports describing favorable outcomes after revascularization procedures.^5,6,10^ Importantly, no statistically significant difference was observed between DRIL and PAI in symptom relief, complication rates, or primary patency.

These findings were particularly relevant given that DRIL has traditionally been regarded as the gold standard for severe DASS,^1,6,14^ largely on the basis of cumulative institutional experience rather than comparative evidence. Our pooled data did not identify a statistically significant advantage of DRIL over PAI, although the strength of this observation is substantially limited by the small, retrospective, and heterogenous nature of the included studies.

DASS represents a complex hemodynamic disorder resulting from preferential diversion of blood flow through the arteriovenous access in the setting of limited distal arterial reserve.^1,2,15,16^ Although physiological steal is common, only a subset of patients develop clinically significant ischemia requiring intervention.^3,4^

The high pooled symptom resolution rate observed in this analysis aligns with historical series demonstrating that surgical correction reliably restores distal perfusion.^5,6,10,17^ Notably, DRIL achieved a 91% resolution rate, consistent with previously reported midterm outcomes,^6,14^ while PAI demonstrated a 93% resolution rate, similar to earlier prospective experiences.^7,8^

The absence of statistically significant differences between techniques should be interpreted with caution. A key limitation across studies is confounding by indication: DRIL was typically used in more severe ischemia (rest pain, tissue loss, and motor dysfunction), whereas PAI was applied in less severe or anatomically selected cases. This nonrandom allocation introduces bias that cannot be corrected without patient-level data. Therefore, similar pooled outcomes between DRIL and PAI may reflect case-mix imbalance rather than true equivalence. This issue is further reinforced by the lack of standardized selection protocols and notable intersurgeon variability reported across studies.

DRIL was originally described to restore distal perfusion while maintaining access function and has demonstrated durable midterm patency and symptom relief.^6^ Its physiological rationale—bypass of the inflow segment combined with distal arterial ligation—directly addresses the pathophysiology of steal syndrome.^15,17^ For these reasons, DRIL has been widely adopted and incorporated into contemporary practice algorithms.^18^

However, the designation of DRIL as the gold standard has not been supported by randomized comparisons. Previous systematic reviews have focused on single techniques rather than comparative effectiveness.^14^ By contrast, the present analysis directly compared data across reconstructive strategies and did not demonstrate superiority of DRIL over PAI in clinical improvement, safety, or patency. Thus, while DRIL remains a highly effective and well-established intervention, the current evidence base did not justify an unequivocal hierarchical preference over PAI.

PAI was introduced as an alternative strategy designed to rebalance inflow dynamics without sacrificing the native axial artery.^7,8^ By shifting inflow proximally to a larger caliber artery, the aim of PAI is to reduce distal hypoperfusion while maintaining adequate access flow.

Early reports of PAI demonstrated encouraging clinical success,^7,8^ and more recent series have reported favorable symptom relief and acceptable patency.^10^ In our pooled analysis, no statistically significant difference between PAI and DRIL was detected across major end points. These findings reinforce the concept that PAI was not merely a secondary option but a legitimate reconstructive strategy, particularly in patients where ligation of the native artery is undesirable. From a practical perspective, procedure selection should incorporate anatomical considerations, access history, and patient comorbidities rather than adherence to a single standardized approach.

The pooled complication rate was low and did not differ significantly between techniques. This observation aligned with previous series reporting acceptable morbidity profiles for both DRIL and PAI.^5,6,10^ While DRIL entails bypass construction and arterial ligation and PAI typically requires prosthetic conduit implantation, neither strategy demonstrated a technique-driven increase in postoperative complications in pooled analysis.

Primary patency outcomes did not differ significantly between groups. Although heterogeneity was substantial, this likely reflects variability in follow-up duration, patency definitions, and access types across cohorts.^19^ Importantly, no statistically significant difference in patency was observed between DRIL and PAI, supporting the conclusion that both techniques were able to preserve dialysis access function effectively.

Moderate heterogeneity was observed for symptom resolution and substantial heterogeneity for primary patency. Such variability is expected in surgical literature, where technique selection and patient characteristics differ between institutions.^10,17^ Exploratory meta-regression did not demonstrate significant associations between treatment effect and publication year or study size, arguing against strong temporal trends or small-study effects.

Radial plot analysis did not identify extreme outliers driving heterogeneity, suggesting that dispersion reflected genuine interstudy variability rather than statistical instability.

This analysis represented one of the first comparative syntheses specifically evaluating DRIL and PAI in DASS. Strengths include logit-transformed pooling, subgroup analysis by technique, trim-and-fill assessment of publication bias, and influence diagnostics.

However, several important limitations must be acknowledged. First, all included studies were observational and predominantly retrospective, with most judged to be at serious risk of bias according to the ROBINS-I instrument, primarily due to confounding by indication and nonrandom allocation of surgical technique. Second, the small number of studies included in subgroup analyses substantially limits the statistical power and reliability of meta-regression, funnel plot asymmetry assessment, Egger test, and trim-and-fill analysis—all of which require a larger number of studies to yield robust inferences. Third, heterogeneity in outcome definitions, particularly for primary patency, and variability in follow-up duration across cohorts limit the clinical interpretability of pooled estimates. Fourth, the absence of patient-level data precluded adjustment for ischemia severity, access type, or comorbidity burden, all of which are likely to influence technique selection and outcomes. Accordingly, the absence of a statistically significant difference between DRIL and PAI should not be interpreted as evidence of equivalence, and conclusions must be considered hypothesis generating rather than definitive.

In the absence of a universally superior technique, surgical decision making in DASS should be guided by individual anatomical and clinical factors, supported by available evidence and expert consensus.^1,2,18^

DRIL is preferred when the native distal artery is suitable for bypass, in patients with severe ischemia (Tordoir 3–4), and when an adequate autogenous vein (≥3 mm) is available. It is particularly indicated in high-flow brachiocephalic or brachiobasilic AVF requiring direct distal revascularization.^6,14^

PAI is indicated when distal arteries are inadequate for ligation (small caliber, calcification, or absent radial artery), when arterial continuity must be preserved, or when no suitable vein is available. It is also useful in prosthetic arteriovenous graft‑related DASS and in patients with distal arterial occlusive disease.^7,8,10^

RUDI may be considered in high-flow brachiocephalic AVF with feasible distal inflow redistribution, especially in younger patients with moderate ischemia (Tordoir 2–3) and adequate distal vessels, when ligation or prosthetic grafting is undesirable.^13^

This framework is based on observational data and expert opinion rather than randomized trials.^1^ Final decisions should incorporate comorbidities, life expectancy, future access needs, and patient preferences. Preoperative hemodynamic assessment and vascular imaging are strongly recommended to optimize outcomes.^5,18^

Flow-reduction techniques, such as banding, show inconsistent results, with risks of thrombosis and persistent ischemia, although intraoperative flow monitoring has been associated with improved outcomes.^20,21^

Given that no statistically significant difference in effectiveness or safety was detected between techniques—within the limitations of the available observational evidence—surgical decision making in DASS should be individualized on the basis of anatomical, clinical, and patient-specific factors. While DRIL remains a cornerstone procedure with well-documented durability,^6,14^ PAI may represent a reasonable alternative in appropriately selected patients, although this cannot be confirmed without higher quality comparative data.^7,8,10^

The current evidence base is insufficient to support a single universally preferred technique. Future research priorities include prospective multicenter registries with systematic stratification by DASS severity, access type, and ischemia etiology; risk-adjusted comparative analyses incorporating patient-level data; and the development of validated anatomical and hemodynamic decision frameworks.

Although DRIL has been considered the gold standard for DASS, this meta-analysis did not demonstrate a statistically significant difference between DRIL and PAI in symptom resolution, complications, or primary patency. These findings must be interpreted cautiously given the small number of retrospective studies, serious risk of bias, and substantial heterogeneity. They should not be interpreted as evidence of clinical equivalence but rather as hypothesis generating. In the absence of randomized comparative data, treatment should be individualized, and well-designed prospective studies are urgently needed to inform evidence-based surgical decision making.

## Supplementary Material

## Data Availability

Original data generated for the study will be made available upon reasonable request to the corresponding author. Data Type: Aggregated Data, Observational Data, and Published Material. Reason for Restricted Access: Data are available from the corresponding author upon reasonable request and do not require restricted repository access.

## Supplemental Material

Supplemental Figure 1. Galbraith (radial) plots for between-study heterogeneity.

## References

[1] Malik J Tuka V Kasalova Z, . Understanding the dialysis access steal syndrome. A review of the etiologies, diagnosis, prevention and treatment strategies. J Vasc Access. 2008;9(3):155–166. PMID: 18850575 18850575

[2] Tordoir JH Dammers R van der Sande FM. Upper extremity ischemia and hemodialysis vascular access. Eur J Vasc Endovasc Surg. 2004;27(1):1–5. doi:10.1016/j.ejvs.2003.10.007 14652830

[3] Papasavas PK Reifsnyder T Birdas TJ Caushaj PF Leers S. Prediction of arteriovenous access steal syndrome utilizing digital pressure measurements. Vasc Endovascular Surg. 2003;37(3):179–184. doi:10.1177/153857440303700304 12799726

[4] Mascia S Spiezia S Assanti A, . Ischemic steal syndrome in a hemodialysis patient: the roles of Doppler ultrasonography and dynamic Doppler studies in diagnosis and treatment selection. J Ultrasound. 2010;13(3):104–106. doi:10.1016/j.jus.2010.09.003 PMC3552993 23396797

[5] Leake AE Winger DG Leers SA Gupta N Dillavou ED. Management and outcomes of dialysis access-associated steal syndrome. J Vasc Surg. 2015;61(3):754–760. doi:10.1016/j.jvs.2014.10.038 25499703

[6] Huber TS Brown MP Seeger JM Lee WA. Midterm outcome after the distal revascularization and interval ligation (DRIL) procedure. J Vasc Surg. 2008;48(4):926–933. doi:10.1016/j.jvs.2008.05.028 18639413

[7] Thermann F Wollert U Ukkat J Dralle H. Proximalization of the arterial inflow (PAI) in patients with dialysis access-induced ischemic syndrome: first report on long-term clinical results. J Vasc Access. 2010;11(2):143–149. doi:10.1177/112972981001100211 20155715

[8] Zanow J Kruger U Scholz H. Proximalization of the arterial inflow: a new technique to treat access-related ischemia. J Vasc Surg. 2006;43(6):1216–1221. doi:10.1016/j.jvs.2006.01.025 16765242

[9] Knox RC Berman SS Hughes JD Gentile AT Mills JL. Distal revascularization–interval ligation: a durable and effective treatment for ischemic steal syndrome after hemodialysis access. J Vasc Surg. 2002;36:250–256. doi:10.1067/mva.2002.125025 12170205

[10] Gupta N Yuo TH Konig G IV, . Treatment strategies of arterial steal after arteriovenous access. J Vasc Surg. 2011;54(1):162–167. doi:10.1016/j.jvs.2010.10.134 21276691

[11] Alie-Cusson FS Bhat K Ramchandani J Steerman SN Dexter DJ Panneton JM. Distal revascularization and interval ligation for the management of dialysis access steal syndrome. Ann Vasc Surg. 2021;74:29–35. doi:10.1016/j.avsg.2021.01.102 33819594

[12] Bachmann C Parker M Sardana T Ramsey L Hashemi H. Proximalization of arterial inflow for treatment of hemodialysis access-induced distal ischemia. J Vasc Surg. 2024;80:1090–1096. doi:10.1016/j.jvs.2024.05.045 38909917

[13] Kordzadeh A Garzon LAN Parsa AD. Revision using distal inflow for the treatment of dialysis access steal syndrome: a systematic review. Ann Vasc Dis. 2018;11(4):473–478. doi:10.3400/avd.ra.18-00051 PMC6326047 30637001

[14] Kordzadeh A Parsa AD. A systematic review of distal revascularization and interval ligation for the treatment of vascular access-induced ischemia. J Vasc Surg. 2019;70(4):1364–1373. doi:10.1016/j.jvs.2019.02.060 31153703

[15] Papadoulas SI Kouri N Tsimpoukis A, . Treatment options for dialysis access steal syndrome. Kardiochir Torakochirurgia Pol. 2022;19(3):141–145. doi:10.5114/kitp.2022.119762 PMC9574582 36268490

[16] Stolic R Mijailovic M Cvetkovic A Stanojevic M Stolic DZ Cvetkovic D. Distal ischemia induced by vascular access for hemodialysis - a case report. Hippokratia. 2012;16(4):375–377. PMID: 23935322 PMC3738617 23935322

[17] Allon M Cutter GR Young CJ. Vascular access-related distal ischemia requiring intervention: frequency, risk factors, and consequences. Clin J Am Soc Nephrol. 2023;18(12):1592–1598. doi:10.2215/CJN.0000000000000310 PMC10723913 37707801

[18] Lok CE Huber TS Lee T, . KDOQI clinical practice guideline for vascular access: 2019 update. Am J Kidney Dis. 2020;75(4 suppl 2):S1–S164. doi:10.1053/j.ajkd.2019.12.001 32778223

[19] Sidawy AN Gray R Besarab A, . Recommended standards for reports dealing with arteriovenous hemodialysis accesses. J Vasc Surg. 2002;35(3):603–610. doi:10.1067/mva.2002.122025 11877717

[20] Miller GA Goel N Friedman A, . The MILLER banding procedure for dialysis-associated steal syndrome. Kidney Int. 2010;77(4):359–366. doi:10.1038/ki.2009.461 20010547

[21] Zanow J Petzold K Petzold M Krueger U Scholz H. Flow reduction in high-flow arteriovenous access using intraoperative flow monitoring. J Vasc Surg. 2006;44(6):1273–1278. doi:10.1016/j.jvs.2006.08.010 17145429

