# Supplementary material for: Is Distal Revascularization with Interval Ligation Still the Gold Standard? A Comparative Meta-Analysis and Meta-Regression of Surgical Treatments of Dialysis Access–Associated Steal Syndrome
Source: Kidney360. 2026 Jun 16;7(9):2087–101. doi: 10.34067/KID.0000001252 (PMC13641455; doi:10.34067/KID.0000001252)
Supplement: Supplementary file 1 [file kidney360-7-2087-s001.pdf]

ASN American Society of Nephrology · JASN Journal of the American Society of Nephrology · KIDNEY360 Accessing Our World From Every Angle · CJASN Clinical Journal of the American Society of Nephrology

ASN Journal Disclosure Form

As per ASN journal policy, I have disclosed any financial relationships or commitments I have held in the past 36 months as included below. I have listed my Current Employer below to indicate there is a relationship requiring disclosure. If no relationship exists, my Current Employer is not listed.

S. Asensio reports the following:
Employer: Hospital Clínico Universitario de Valladolid

I understand that the information above will be published within the journal article, if accepted, and that failure to comply and/or to accurately and completely report the potential financial conflicts of interest could lead to the following: 1) Prior to publication, article rejection, or 2) Post-publication, sanctions ranging from, but not limited to, issuing a correction, reporting the inaccurate information to the authors' institution, banning authors from submitting work to ASN journals for varying lengths of time, and/or retraction of the published work.

Name: Sergio Asensio
Manuscript ID: K360-2026-000310R3
Manuscript Title: Is DRIL still the gold standard? A comparative meta-analysis and meta-regression of surgical treatments for dialysis-access–associated steal syndrome
Date of Completion: May 13, 2026
Disclosure Updated Date: May 13, 2026

ASN Journal Disclosure Form

As per ASN journal policy, I have disclosed any financial relationships or commitments I have held in the past 36 months as included below. I have listed my Current Employer below to indicate there is a relationship requiring disclosure. If no relationship exists, my Current Employer is not listed.

M. Caja reports the following:
Employer: Hospital Clínico Universitario de Valladolid

I understand that the information above will be published within the journal article, if accepted, and that failure to comply and/or to accurately and completely report the potential financial conflicts of interest could lead to the following: 1) Prior to publication, article rejection, or 2) Post-publication, sanctions ranging from, but not limited to, issuing a correction, reporting the inaccurate information to the authors' institution, banning authors from submitting work to ASN journals for varying lengths of time, and/or retraction of the published work.

Name: Marina Jiménez Caja
Manuscript ID: K360-2026-000310R4
Manuscript Title: Is DRIL still the gold standard? A comparative meta-analysis and meta-regression of surgical treatments for dialysis-access–associated steal syndrome
Date of Completion: May 31, 2026
Disclosure Updated Date: May 11, 2026

ASN American Society of Nephrology
JASN Journal of the American Society of Nephrology
KIDNEY360 Accessing Our World From Every Angle
CJASN Clinical Journal of the American Society of Nephrology

ASN Journal Disclosure Form

As per ASN journal policy, I have disclosed any financial relationships or commitments I have held in the past 36 months as included below. I have listed my Current Employer below to indicate there is a relationship requiring disclosure. If no relationship exists, my Current Employer is not listed.

C. de la Torre Casaseca has nothing to disclose.

I understand that the information above will be published within the journal article, if accepted, and that failure to comply and/or to accurately and completely report the potential financial conflicts of interest could lead to the following: 1) Prior to publication, article rejection, or 2) Post-publication, sanctions ranging from, but not limited to, issuing a correction, reporting the inaccurate information to the authors' institution, banning authors from submitting work to ASN journals for varying lengths of time, and/or retraction of the published work.

Name: Clara de la Torre Casaseca
Manuscript ID: K360-2026-000310R3
Manuscript Title: Is DRIL still the gold standard? A comparative meta-analysis and meta-regression of surgical treatments for dialysis-access–associated steal syndrome
Date of Completion: May 12, 2026
Disclosure Updated Date: May 12, 2026

ASN American Society of Nephrology
JASN Journal of the American Society of Nephrology
KIDNEY360 Accessing Our World From Every Angle
CJASN Clinical Journal of the American Society of Nephrology

ASN Journal Disclosure Form

As per ASN journal policy, I have disclosed any financial relationships or commitments I have held in the past 36 months as included below. I have listed my Current Employer below to indicate there is a relationship requiring disclosure. If no relationship exists, my Current Employer is not listed.

M. DEL RIO-SOLA has nothing to disclose.

I understand that the information above will be published within the journal article, if accepted, and that failure to comply and/or to accurately and completely report the potential financial conflicts of interest could lead to the following: 1) Prior to publication, article rejection, or 2) Post-publication, sanctions ranging from, but not limited to, issuing a correction, reporting the inaccurate information to the authors' institution, banning authors from submitting work to ASN journals for varying lengths of time, and/or retraction of the published work.

Name: MARIA LOURDES DEL RIO-SOLA
Manuscript ID: K360-2026-000310R1
Manuscript Title: s DRIL still the gold standard? A comparative meta-analysis and meta-regression of surgical treatments for dialysis-access–associated steal syndrome
Date of Completion: April 15, 2026
Disclosure Updated Date: April 15, 2026

ASN American Society of Nephrology | JASN Journal of the American Society of Nephrology | KIDNEY360 Accessing Our World From Every Angle | CJASN Clinical Journal of the American Society of Nephrology

ASN Journal Disclosure Form

As per ASN journal policy, I have disclosed any financial relationships or commitments I have held in the past 36 months as included below. I have listed my Current Employer below to indicate there is a relationship requiring disclosure. If no relationship exists, my Current Employer is not listed.

S. Fernández reports the following:
Employer: Hospital Clínico Universitario de Valladolid

I understand that the information above will be published within the journal article, if accepted, and that failure to comply and/or to accurately and completely report the potential financial conflicts of interest could lead to the following: 1) Prior to publication, article rejection, or 2) Post-publication, sanctions ranging from, but not limited to, issuing a correction, reporting the inaccurate information to the authors' institution, banning authors from submitting work to ASN journals for varying lengths of time, and/or retraction of the published work.

Name: Sandra Pérez Fernández
Manuscript ID: K360-2026-000310R4
Manuscript Title: Is DRIL still the gold standard? A comparative meta-analysis and meta-regression of surgical treatments for dialysis-access–associated steal syndrome
Date of Completion: May 31, 2026
Disclosure Updated Date: April 14, 2026
