# Supplementary material for: Is Distal Revascularization with Interval Ligation Still the Gold Standard? A Comparative Meta-Analysis and Meta-Regression of Surgical Treatments of Dialysis Access–Associated Steal Syndrome
Source: Kidney360. 2026 Jun 16;7(9):2087–101. doi: 10.34067/KID.0000001252 (PMC13641455; doi:10.34067/KID.0000001252)
Supplement: Supplementary file 2 [file kidney360-7-2087-s002.pdf]

Supplemental Figure 1. Galbraith (Radial) Plots for Between-Study Heterogeneity. (A) Symptom resolution. (B) Postoperative complications. (C) Primary patency. Galbraith (radial) plots were constructed to assess between-study heterogeneity and identify potential outlier studies influencing pooled estimates for each outcome. Logit-transformed effect sizes are plotted against the reciprocal of their standard errors, with study weights proportional to inverse variance. The central line represents the pooled random-effects estimate, and the dashed lines indicate the 95% confidence limits. Studies located outside the confidence boundaries may contribute disproportionately to overall heterogeneity.

A)

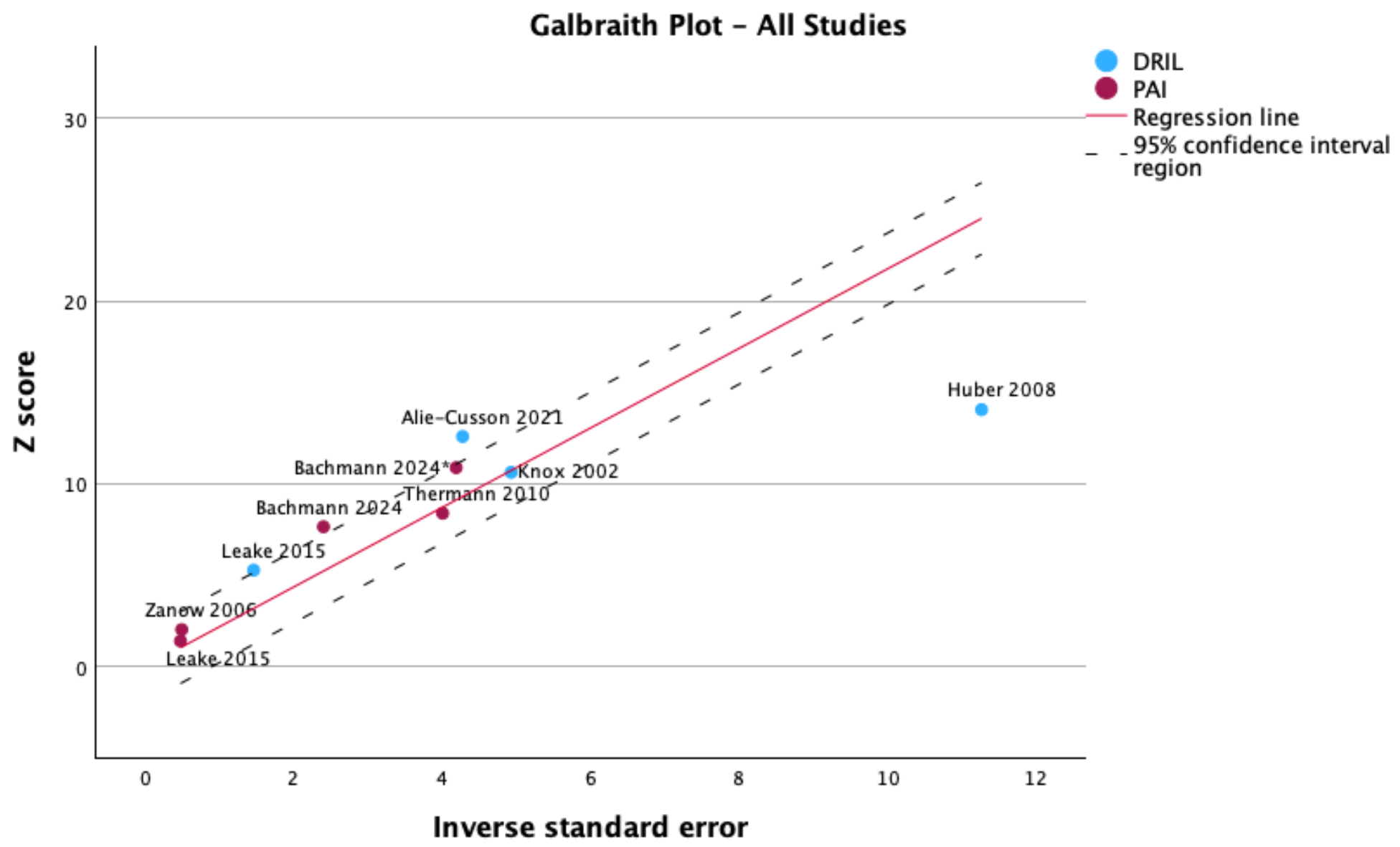

B)

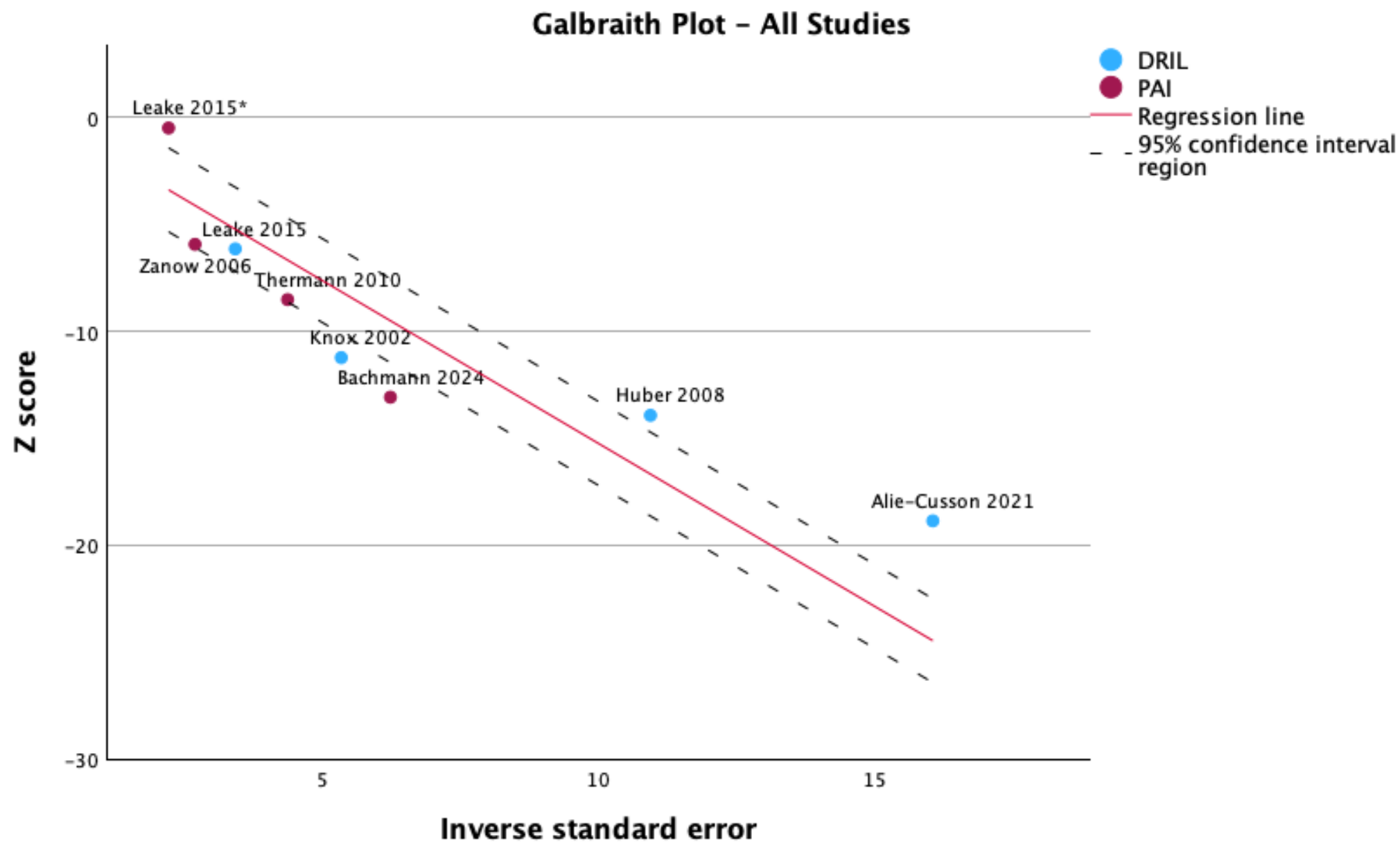

C)

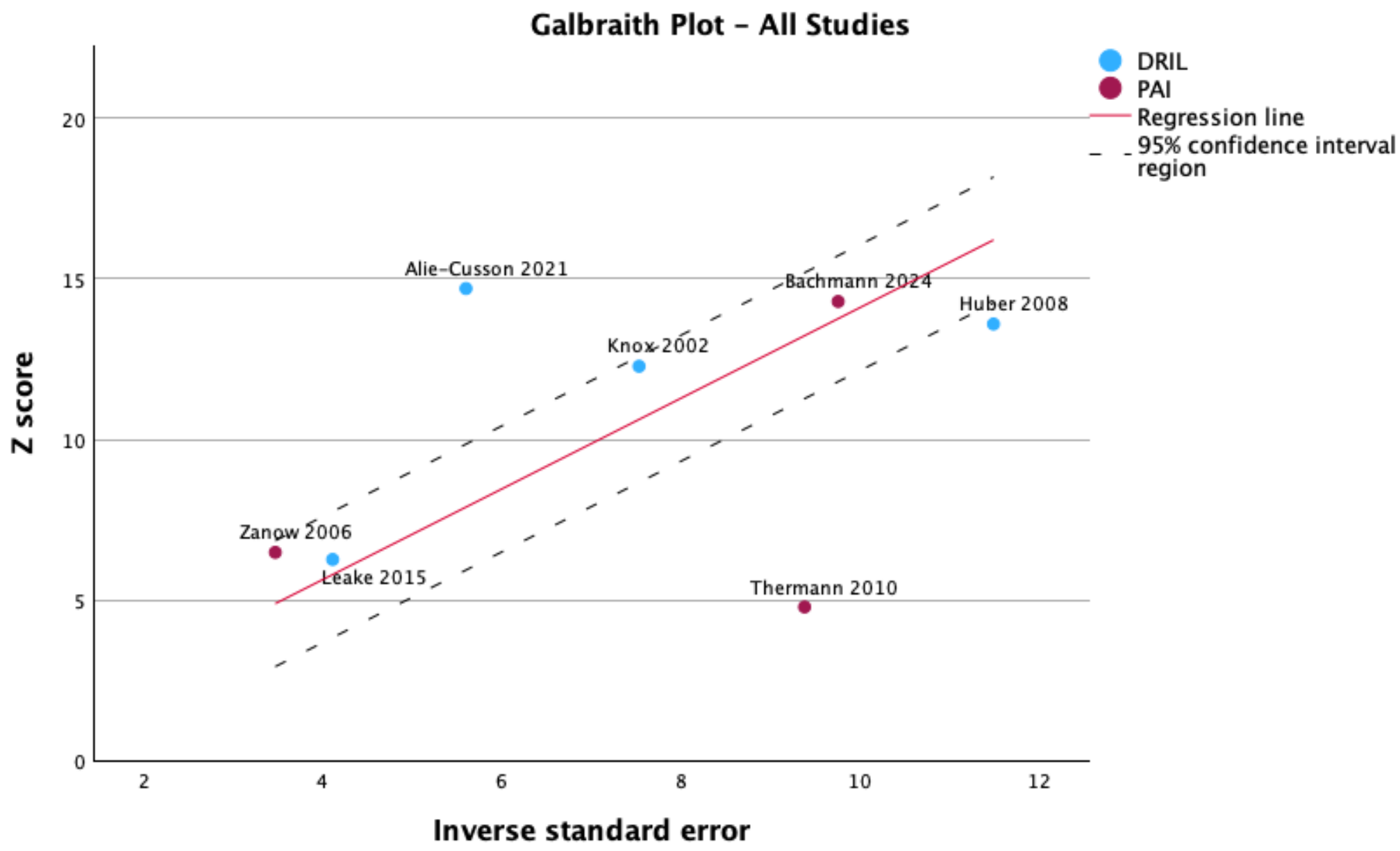
Galbraith Plot - All Studies
DRIL
PAI
Regression line
95% confidence interval region
Z score
Inverse standard error
Alie-Cusson 2021
Bachmann 2024
Huber 2008
Knox 2002
Zanow 2006
Leake 2015
Thermann 2010
0
5
10
15
20
2
4
6
8
10
12

Supplemental Appendix A

Complete Database Search Strategies

The following search strategies were used to identify eligible studies for this systematic review and meta-analysis. All searches were conducted on January 15, 2025. Searches were performed independently by two reviewers and combined using appropriate Boolean operators. No date restrictions were applied (searches covered from database inception to the search date). Language restrictions were not applied at the search stage.

The search strategy was designed to maximize sensitivity while maintaining specificity, combining Medical Subject Headings (MeSH) with free-text terms to capture variations in terminology across the literature.

1. PubMed / MEDLINE

Database: PubMed (National Library of Medicine) | Search date: January 15, 2025 | Interface: pubmed.ncbi.nlm.nih.gov

```
#1  "Steal Syndrome"[MeSH] OR "dialysis access steal"[tiab] OR "access-induced
ischemia"[tiab]
    OR "haemodialysis steal"[tiab] OR "hemodialysis steal"[tiab]
    OR "arteriovenous steal"[tiab] OR "vascular access steal"[tiab]
    OR "DASS"[tiab] OR "HAIDI"[tiab]
    OR "access-associated steal"[tiab] OR "ischemic steal syndrome"[tiab]

#2  "Arteriovenous Fistula"[MeSH] OR "arteriovenous fistula"[tiab]
    OR "arteriovenous graft"[tiab] OR "hemodialysis access"[tiab]
    OR "haemodialysis access"[tiab] OR "vascular access"[tiab]
    OR "dialysis access"[tiab]

#3  "distal revascularization interval ligation"[tiab] OR "DRIL"[tiab]
    OR "proximalization of arterial inflow"[tiab] OR "PAI"[tiab]
    OR "revision using distal inflow"[tiab] OR "RUDI"[tiab]
    OR "proximal arterial inflow"[tiab] OR "distal inflow revision"[tiab]

#4  #1 AND #2

#5  #1 AND #3

#6  #4 OR #5

Final search: #6
Filters: None
```

**Search date**	January 15, 2025
**Records retrieved**	298
**After deduplication**	Combined with other databases

2. Embase

Database: Embase (Elsevier) | Search date: January 15, 2025 | Interface: embase.com

```
#1  'steal syndrome'/exp OR 'dialysis access steal syndrome':ab,ti
    OR 'hemodialysis access steal':ab,ti OR 'haemodialysis access steal':ab,ti
    OR 'vascular access steal':ab,ti OR 'arteriovenous steal':ab,ti
    OR 'access induced ischemia':ab,ti OR 'access associated steal':ab,ti
    OR 'DASS':ab,ti OR 'HAIDI':ab,ti OR 'ischemic steal':ab,ti

#2  'arteriovenous fistula'/exp OR 'arteriovenous fistula':ab,ti
    OR 'hemodialysis access'/exp OR 'hemodialysis access':ab,ti
    OR 'dialysis access':ab,ti OR 'arteriovenous graft':ab,ti
    OR 'vascular access for hemodialysis':ab,ti

#3  'distal revascularization interval ligation':ab,ti OR 'DRIL':ab,ti
    OR 'proximalization of arterial inflow':ab,ti OR 'PAI':ab,ti
    OR 'revision using distal inflow':ab,ti OR 'RUDI':ab,ti
    OR 'proximal arterial inflow':ab,ti

#4  #1 AND #2

#5  #1 AND #3

#6  #4 OR #5

Final search: #6
Filters: None
```

**Search date**	January 15, 2025
**Records retrieved**	241
**After deduplication**	Combined with other databases

3. Cochrane Library (CENTRAL)

Database: Cochrane Central Register of Controlled Trials (CENTRAL) | Search date: January 15, 2025 | Interface: cochranelibrary.com

```
#1  MeSH descriptor: [Steal Syndrome] explode all trees
    OR "dialysis access steal" (Word variations have been searched)
    OR "hemodialysis steal" (Word variations have been searched)
    OR "haemodialysis steal" (Word variations have been searched)
    OR "arteriovenous steal" (Word variations have been searched)
    OR "access induced ischemia" (Word variations have been searched)
    OR "DASS" (Word variations have been searched)
    OR "HAIDI" (Word variations have been searched)

#2  MeSH descriptor: [Arteriovenous Fistula] explode all trees
    OR "arteriovenous fistula" (Word variations have been searched)
    OR "hemodialysis access" (Word variations have been searched)
    OR "dialysis access" (Word variations have been searched)
```

```
    OR "vascular access" (Word variations have been searched)

#3  "distal revascularization interval ligation" (Word variations have been
searched)
    OR "DRIL" (Word variations have been searched)
    OR "proximalization of arterial inflow" (Word variations have been searched)
    OR "PAI" (Word variations have been searched)
    OR "revision using distal inflow" (Word variations have been searched)
    OR "RUDI" (Word variations have been searched)

#4  #1 AND #2

#5  #1 AND #3

#6  #4 OR #5

Final search: #6
Filters: None
```

**Search date**	January 15, 2025
**Records retrieved**	62
**After deduplication**	Combined with other databases

4. Web of Science (Core Collection)

Database: Web of Science Core Collection (Clarivate Analytics) | Search date: January 15, 2025 | Interface: webofscience.com

```
TS = ("steal syndrome" OR "dialysis access steal" OR "haemodialysis steal"
      OR "hemodialysis steal" OR "arteriovenous steal" OR "vascular access
steal"
      OR "access-induced ischemia" OR "access associated steal"
      OR "DASS" OR "HAIDI" OR "ischemic steal syndrome")

AND

TS = ("arteriovenous fistula" OR "arteriovenous graft" OR "hemodialysis access"
      OR "haemodialysis access" OR "dialysis access" OR "vascular access")

OR

TS = ("distal revascularization interval ligation" OR "DRIL"
      OR "proximalization of arterial inflow" OR "PAI"
      OR "revision using distal inflow" OR "RUDI")

Filters: None
```

**Search date**	January 15, 2025
**Records retrieved**	41

**After deduplication**	Combined with other databases

5. Summary of Search Results

Database	Records Retrieved	Duplicates	After Deduplication
PubMed / MEDLINE	298	—	—
Embase	241	—	—
Cochrane Library (CENTRAL)	62	—	—
Web of Science	41	—	—
Manual search of references	18	—	18
**Total**	**660**	**148**	**512**

6. Additional Information

Manual search: In addition to the electronic database searches, the reference lists of all full-text articles assessed for eligibility were manually reviewed to identify additional relevant publications not captured through database searching. This manual search yielded 18 additional records.

PROSPERO registration: The review protocol was prospectively registered in the International Prospective Register of Systematic Reviews (PROSPERO; registration number CRD420261329250) prior to data extraction and study selection.
